# Effective Connectivity Study Guiding the Neuromodulation Intervention in Figurative Language Comprehension Using Optical Neuroimaging

**DOI:** 10.1155/2020/8882207

**Published:** 2020-10-06

**Authors:** Tania Alexandra Couto, Shiyang Xu, Paulo Armada da Silva, Chenggang Wu, Karl Neergaard, Meng-Yun Wang, Juan Zhang, Yutao Xiang, Zhen Yuan

**Affiliations:** ^1^Centre for Cognitive and Brain Sciences, University of Macau, Macau S.A.R., China; ^2^Faculty of Health Sciences, University of Macau, Macau S.A.R.., China; ^3^Faculty of Human Kinetics, University of Lisbon, Lisbon, Portugal; ^4^Faculty of Education, University of Macau, Macau S.A.R., China; ^5^School of Education, Shanghai International Studies University, Shanghai, China

## Abstract

The current study is aimed at establishing links between brain network examination and neural plasticity studies measured by optical neuroimaging. Sixteen healthy subjects were recruited from the University of Macau to test the Granger Prediction Estimation (GPE) method to investigate brain network connectivity during figurative language comprehension. The method is aimed at mapping significant causal relationships across language brain networks, captured by functional near-infrared spectroscopy measurements (fNIRS): (i) definition of regions of interest (ROIs) based on significant channels extracted from spatial activation maps; (ii) inspection of significant causal relationships in temporal resolution, exploring the experimental task agreement; and (iii) early identification of stronger causal relationships that guide neuromodulation intervention, targeting impaired connectivity pathways. Our results propose top-down mechanisms responsible for perceptive-attention engagement in the left anterior frontal cortex and bottom-up mechanism in the right hemispheres during the semantic integration of figurative language. Moreover, the interhemispheric directional flow suggests a right hemisphere engagement in decoding unfamiliar literal sentences and fine-grained integration guided by the left hemisphere to reduce ambiguity in meaningless words. Finally, bottom-up mechanisms seem activated by logographic-semantic processing in literal meanings and memory storage centres in meaningless comprehension. To sum up, our main findings reveal that the Granger Prediction Estimation (GPE) integrated strategy proposes an effective link between assessment and intervention, capable of enhancing the efficiency of the treatment in language disorders and reducing the neuromodulation side effects.

## 1. Introduction

The neuroimaging paradigm has been changing, and a new analysis has emerged to facilitate the understanding of the brain mechanisms. The first functional neuroimaging trend relies on brain function identification based on brain activation between different brain regions [1], while the second trend has emerged from functional connectivity measures between different brain regions in a single brain. Finally, the third and most recent trend suggests between-brain connectivity. In more detail, functional connectivity studies rely on correlation analysis [2], while effective connectivity studies are conducted based on causal algorithms. The causal analysis adds more accuracy to the connectivity study, which allows us to predict how one brain region of interest may influence another one. In this sense, the current study proposes a Granger Prediction Estimation (GPE) method to address the potential of causal analysis and its application to neuroimaging and neuromodulation intervention. More particularly, GPE is a well-established method in econometrics that has been applied to the neuroimaging field, specifically to connectivity data. The technique relies on the usefulness of prediction of the current values in one specific brain region's signals from the past values in other relevant brain regions. Besides, another gap pointed out by the scientific community relies on the limited resolution captured by the near-infrared light. Traditionally, the fNIRS measurements were not usually well addressed by frequency domain in causal analyses, and, so far, no studies have been reported investigating dynamic brain networks applied to time-series resolution inspected by functional near-infrared spectroscopy. Importantly, the previous multivariate autoregressive model seems to address a time-frequency resolution; however, limitations need to be carefully revised when the method does not provide any information about oscillations in neural systems, ignoring critical components of a system's dynamics [3].

Consequently, the functional and effective connectivity studies guided by functional near-infrared spectroscopy (NIRS) have been recently described as a promising technique [2, 4] to map language networks and provide a more reliable inspection to guide the neural plasticity studies. Traditionally, the neuromodulation protocols are guided by functional connectivity indexes (such as amplitude asymmetry, coherence, and phase lag) and rarely by effective connectivity. Therefore, the current study is aimed at filling this gap, relying on its potentiality in mapping stronger versus weaker regions of interest and prioritizing the stronger regions of interest during clinical intervention protocols. Further elaborated, the neuromodulation protocols should address firstly the significant brain networks of interest, which elicited stronger influence over another brain network of interest. The brain networks which generate a limited impact during the language comprehension task might be modulated at the second stage. Injured patients and patients who present impaired language abilities, such as people in the Autism Spectrum Disorder (ASD), can highly benefit from these causal mapping during neuromodulation interventions, rarely conducted for clinical populations.

Extensive research has been conducted to reveal the neural resources and brain networks responsible for language comprehension, especially between literal and figurative language in healthy [5] and clinical population [6]. Underlying metaphor comprehension is the uniquely human capacity to resolve ambiguity in phrases such as “family is a harbour,” where conceptual mapping [7, 8] is needed between the target “family” and its source, “harbour.” The methods related to the investigation of metaphor comprehension have a wide range of applications, reaching from the diagnoses of learning deficits [9], untangling impairment due to psychosis [10], to the study of creativity [11].

Traditional versus parallel hypothesis helps us understand their role in the selection or suppression of adequate meanings during the processing of nonliteral language. The traditional hypothesis claims that literal meaning is processed first to facilitate the metaphor processing [8, 12], while the more recently proposed parallel hypothesis states concurrent processing of both meanings [13]. Based on previous evidence, the role of the left inferior frontal gyrus (BA45/47) is understood as relevant for the selection of proper meanings or for the suppression of nonrelevant meanings [14], while the middle temporal gyrus (MTG) and the superior temporal gyrus (STG) represent a highly connected network extended from frontal to parietal structures responsible for the processing of figurative language and complex connotation [15]. Moreover, the integrative role of the left inferior parietal cortex (IPC) and left angular gyrus (AG) seems to be essential for adjusting individual representations into a coherent meaning and context [16, 17]. In general, consistent patterns of activation in frontal, temporal, and parietal networks play a key role in the characterization of figurative and literal language comprehension [5]. However, the extent to which those networks interact and influence each other remains unclear.

To sum up, the Granger Prediction Estimation (GPE) method is proposed for fNIRS data to identify brain networks, explaining how one specific neural mechanism might exert influence on another [18, 19] and illustrate how the causal mappings might be crucial to address the neuromodulation protocols in a healthy or clinical population.

## 2. Materials and Methods

### 2.1. Participants

Sixteen Chinese speakers participated in the current study (8 males and 8 females; mean age: 24.5 years old). None of the participants reported a history of psychiatric or neurological disorders. All participants were right-handed and had a normal or corrected-to-normal vision. They gave informed consent according to the protocol for research ethics of the University of Macau. The sample characteristics are provided in Table 1.

### 2.2. Materials

The stimulus materials consist of three categories of sentences: (1) literal, (2) metaphorical, and (3) meaningless sentences. Each category contains 40 sentences, and the three categories of sentences were matched according to grammar, tense, and syntactic structure. It is noted that the only difference between the three kinds of sentences was that the end words (i.e., critical words) were changed. For example, a metaphorical sentence could be described as “家庭是港湾” (family is a harbour), and an expression for the literal category would be “家庭是责任” (family is a responsibility), whereas the description for a meaningless sentence would be “家庭是床垫” (family is a mattress). In addition, sentence categories were also matched for the number of words. Furthermore, the Chinese words of the three categories of sentences were matched considering the Chinese words' frequency and Chinese words' strokes F′s < 0.3. Only simple sentences were adopted for the present paradigm to keep it consistent among the three conditions. To control other lexical characteristics of the sentences and confirm novelty and figurativeness, online rating was performed in a pilot survey by another group of 20 participants who were asked to rate all 120 sentences on a six-point Likert scale on familiarity, imagery, figurativeness, novelty, content, and meaningfulness. The meaningless sentences were lower in familiarity (F (2, 117) = 51.748, p < 0.001), content (F (2, 117) = 155.302, p < 0.001), and meaningfulness (F (2, 117) = 14.086, p < 0.001), whereas the metaphorical and literal sentences were the same for the three instances. In addition, it was discovered that the metaphorical sentences were also higher in figurativeness (F (2, 117) = 33.717, p < 0.001), novelty (F (2, 117) = 112.742, p < 0.001), and imagery (F (2, 117) = 228.171, p < 0.001) than the literal and meaningfulness ones.

### 2.3. Granger Prediction Estimation (Over Time Series)

The current computational direction relies on bivariate autoregression to understand causality [18]. In other words, the function reveals how current values on time course depend on its past values. For a better prediction, we should understand different parameters.

Firstly, the equation depends on the order that represents the number of the time courses proposed in the prediction, while the coefficient represents vectors defined by the number of the time courses explained by the order parameter. Secondly, *X* represents a function of previous values of *X* (a univariate autoregression), while *Y* represents a function of previous values of *X* (a bivariate autoregressive process). Each equation integrates two coefficient vectors: *a* and *b*; *c* and *d*. Those values can be different among them, which reflects that the influence of *X* on *Y* might be different from the influence of *Y* on *X*. Finally, error terms (exy and eyx) indicate the variance range of the errors for each time point. In other words, it represents the part of the function we cannot predict but is crucial for a realistic prediction.

### 2.4. Dynamic Brain Networks

The previous strategy computes Granger Prediction for one long time segment. However, the next computational step proposes a “successive time segment” approach [18], which enables the understanding of the dynamic changes of the connectivity over time segments influenced by the task events. Additionally, the author recommends that long segments provide a more stable perspective and are less affected by noise. By contrast, the shorter time segments suggested a higher temporal precision, which in turn has a higher sensitivity by identifying transient effects. This dynamic computational direction was chosen consistently to illustrate all the directional relations in metaphor condition, which has revealed significant recruitment of oxygenated haemoglobin and brain activation compared to other conditions.

### 2.5. Behaviour Tasks and fNIRS Recordings

**Figure 1:**
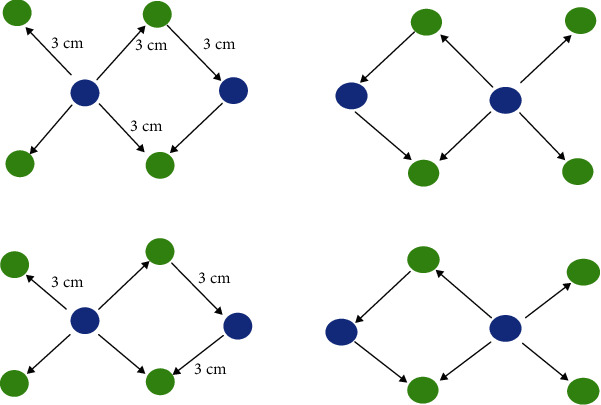
Configuration of laser sources and optical detector pair: 24 channels were generated to cover both the Broca and Wernicke areas and respective contralateral sides.

The participants were instructed to read the sentences that were presented by a single word, in sequence, to decide whether the sentences were meaningful or not. The screen was 80 cm away from the participants. The task contained three conditions: metaphorical, literal, and meaningless sentences. Each condition consisted of 40 trials, and a fixation cross was displayed on the monitor for 500 ms, followed by a sentence presentation during 2200 ms. Each word was presented during 400 ms, and 500 ms was taken for the intervals between the trials. A blank space was introduced for 750 ms before the question mark. Participants needed to provide their meaningfulness judgments by pressing different buttons for various conditions. A continuous-wave (CW) fNIRS setup (CW6 fNIRS system; TechEn Inc., Milford, MA) was used to conduct the current experiment. The system included 8 laser sources and 16 optical detectors. The variances of both oxygenated (HbO) and deoxygenated haemoglobin (HbR) concentrations in the brain were detected by two lights (wavelengths: 690 nm and 830 nm) emitted from every source fibre. Based on a configuration of source-detector, 24 channels were generated to cover both Broca's and Wernicke's areas across the two hemispheres (Figure 1). A 3D magnetic space digitizer, Patriot Digitizer (Polhemus Inc.), was used to measure the 3D spatial information of each optode for each subject. A probabilistic registration method from NIRS-SPM software was used to estimate each channel's corresponding location in the Montreal Neurological Institute (MNI) space [19]. The fNIRS data processing was performed using Homer2_UI (v1.5.2) [20]. The measurements of optical density were first converted to the concentration changes of HbO and HbR at different time points. Then, the raw haemoglobin continuous data was processed by a low-pass filter of 0.2 Hz and subsequently a high-pass filter of 0.015 Hz. An automatic motion artifact detection algorithm from the Homer2 fNIRS processing package was utilized for motion correction [20]. The distance between each source and each detector was 3 cm, and the fNIRS sampling rate was 50 Hz. In this study, only HbO data was analysed due to its high sensitivity. The peak values of run-averaged HbO data were extracted for each channel from each participant for further analysis.

### 2.6. Statistical Analysis

In the current study, ANOVA repeated measures were firstly conducted in a within-subject design, followed by post hoc pairwise comparison tests under the Bonferroni correction to investigate the brain network region of interest (ROI). Secondly, the connectivity interactions among four regions of interest (ROIs) were explored using Granger Prediction Estimation at the first stage: (a) over time series for one sequence of trials (first block) and at the second stage: (b) over specific task periods (such as onset, stimulus duration, and semantic judgment), across the three experimental conditions: metaphor, literal, and meaningless. A *t*-test was conducted across conditions between the times series of each region of interest (ROI) using the leading statistical software IBM SPSS Statistics (2009), after being computed by Granger Prediction Estimation in MATLAB R2012a.

## 3. Results

### 3.1. Behavioural Results

We identified the main effect of sentence type, *F* (2, 30) = 19.271, *p* < 0.001. Multiple comparisons showed that metaphoric sentences exhibited a lower accuracy rate (M 0.577; SD 0.208) than literal (M 0.803; SD 0.143) and meaningless (M 0.879; SD 0.108) sentences (see Table 2).

### 3.2. Regions of Interest (ROIs)

**Figure 2:**
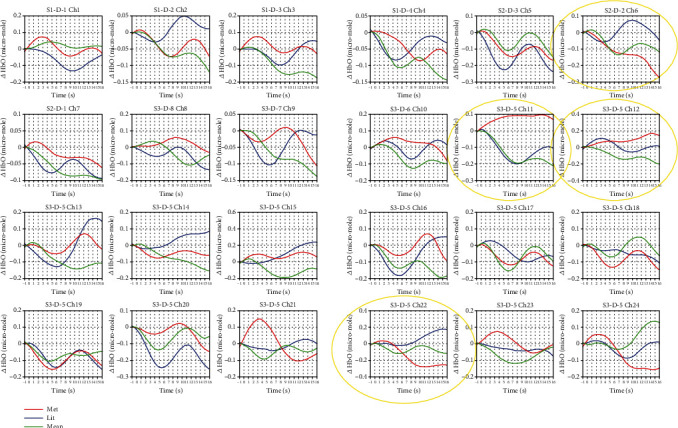
Haemodynamic average changes. Significant differences were only activated in metaphor condition: Broca's area left IFG/channel 06; contralateral side of Broca's area right IFG/channel 11 and channel 12; contralateral side of Wernicke's area right MTG/channel 22 (*p* < 0.05^∗^); and nonsignificant activation Wernicke left MTG/channel 14 (*p* > 0.05).

**Figure 3:**
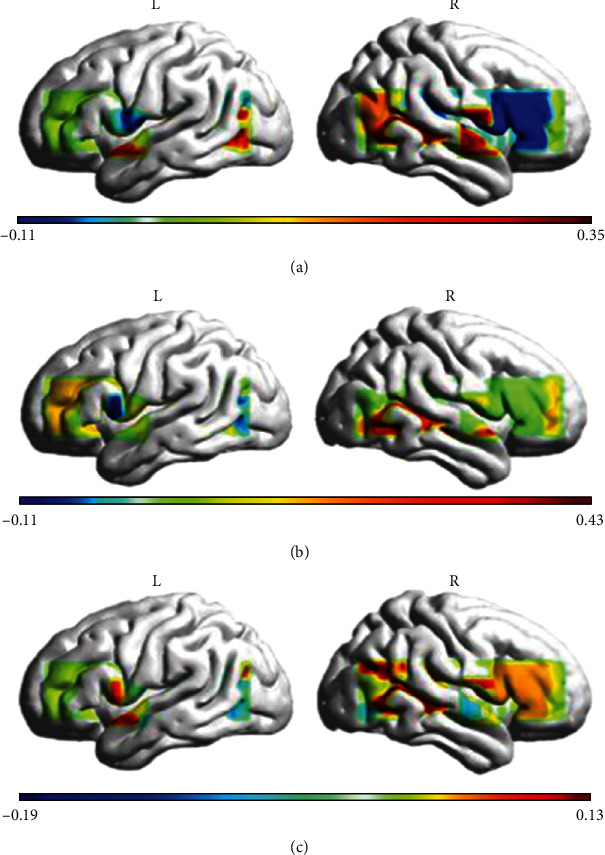
Brain activation study to identify the significant ROIs in the three conditions: (a) metaphor (*p* < 0.05^∗^); (b) literal; (c) meaningless.

The region of interest is defined based on oxygenated haemoglobin and brain activation studies. We generated grand averaged concentration changes in HbO from all channels in the three conditions. Most importantly, to map the brain activation during the processing of various conditions, the HbO images were also obtained from a brain cortex template using the BrainNet Viewer tool [21].  Figure 2 shows the time courses of grand averaged HbO signals for all channels, and Figure 3 presents the brain activation study.  Table 3 illustrates the anatomic labels for all the channels (Brodmann areas and respective MNI coordinates).

A 3 (sentence type : metaphorical, literal, and meaningless) × 24 (number of channels) repeated-measure ANOVA was conducted, in which both variables were within-subject variables. We discovered that the main effect of sentence types was identified in four channels with metaphor condition (channel 6: *F* (2, 30) = 4.102, *p* < 0.035, partial *η*^2^ = 0.215; channel 11: *F* (2, 30) = 4,317, *p* < 0.030, partial *η*^2^ = 0.223; channel 12: *F* (2, 30) = 4,186, *p* < 0.027, partial *η*^2^ = 0.218; and channel 22: *F* (2, 30) = 4.445, *p* < 0.044, partial *η*^2^ = 0.229). In addition, post hoc pairwise comparison tests with Bonferroni correction were performed, which revealed that the metaphorical sentences exhibited significantly higher haemodynamic responses than the literal sentences in channel 6 (*p* < 0.031) and meaningless ones in channel 12 (*p* < 0.035), while no other comparisons reached significance.

### 3.3. GPE over Time Series in Metaphor Comprehension

**Figure 4:**
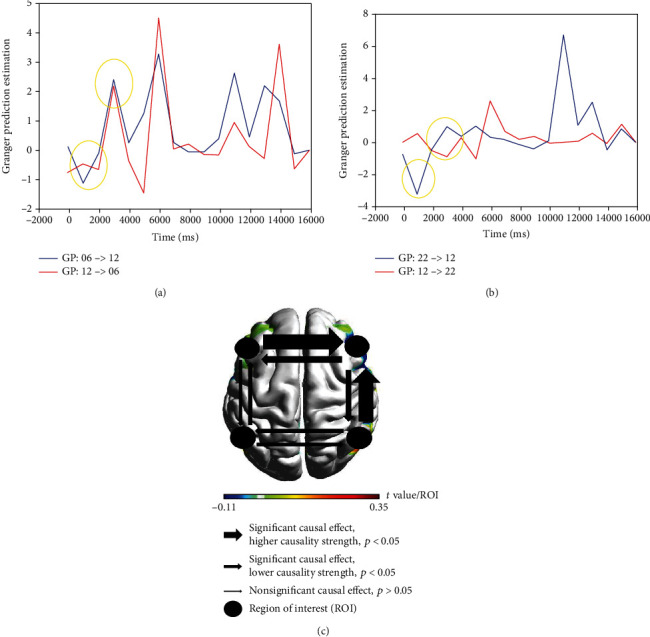
Granger Prediction Estimation generated directional relations measured from HBO2 measurements among significant ROIs in metaphor condition: (a) right Broca/CH12 to left Broca/Ch06 (*p* < 0.041); (b) right Wernicke/Ch22 to right Broca/Ch12 (*p* < 0.021); the first yellow circle represents the onset of the first trial over 500 ms, and the second one represents the stimulus presentation period of the first trial over 2200-2700 ms integrating the semantic judgment; (c) causality strength across ROI (*p* < 0.05^∗^) in metaphor condition during the experimental task, where it illustrated significant causal effect (higher and lower causality strength) versus nonsignificant causal effects.

Granger Prediction estimated directional relations from HbO measurements among four significant ROIs (left Broca/Ch06, left Wernicke/Ch14, right Broca/Ch12, and right Wernicke/Ch22) in metaphor condition during the experimental task (Figure 4). Left Broca and right Wernicke present higher causality strength compared to other ROIs (^∗^*p* < 0.05). Time series causal relations are provided as the *x*-axis, and GPE is given in the left column as the *y*-axis. A significant causal effect (*p* < 0.05) with higher causality strength is illustrated by a larger arrow, while the narrow arrow presents lower causality strength. Nonsignificant causality (*p* > 0.05) is represented by the narrowest arrow, while the brain regions of interest (ROIs) are illustrated by the circle symbol. The *t* value was presented to indicate the activation level elicited by each ROI.

### 3.4. GPE over Time Series in Literal Comprehension

**Figure 5:**
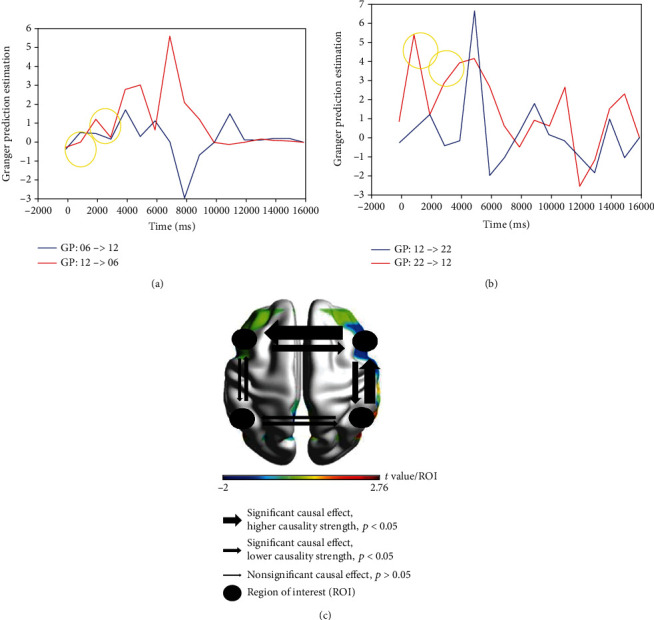
Granger Prediction Estimation generated directional relations measured from HBO2 measurements among significant ROIs in literal condition: (a) right Broca/Ch12 to left Broca/CH06 (*p* < 0.045); (b) right Wernicke/Ch22 to right Broca/Ch14 (*p* < 0.015). The first yellow circle represents the onset of the first trial over 500 ms, and the second one represents the stimulus presentation period of the first trial over 2200-2700 ms interval integrating the semantic judgment period; (c) causality strength across ROI (*p* < 0.05) in the literal condition during the experimental task.

Granger Prediction Estimation generated directional relations measured from HBO2 measurements among four significant ROIs (left Broca/Ch06; left Wernicke/Ch14; right Broca/Ch12; and right Wernicke/Ch22) in literal condition during the experimental task (Figure 5). Right Broca and right Wernicke present higher causality strength compared to other ROIs (^∗^*p* < 0.05). Time series causal relations are provided as the *x*-axis, and GPE is presented in the left column as the *y*-axis. As for the significant causal effect, *p* < 0.05, higher causality strength is illustrated by a larger arrow, while the narrow arrow presents lower causality strength. The narrowest arrow represents nonsignificant causal relationships (*p* > 0.05), and the circle symbol illustrates the brain regions of interest (ROIs). The *t* value was introduced to indicate the activation level elicited by each ROI.

### 3.5. GPE over Time Series in Meaningless Comprehension

**Figure 6:**
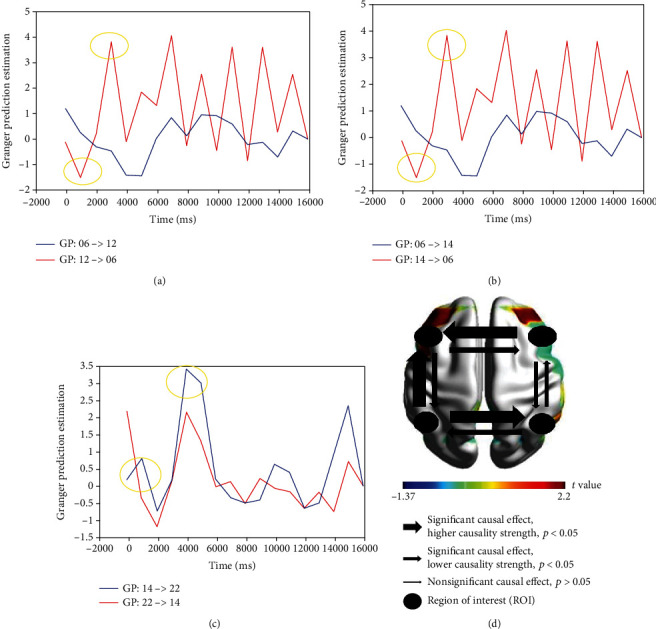
Granger Prediction Estimation generated directional relations measured from HBO2 measurements among significant ROIs in meaningless condition: (a) right Broca/Ch12 to left Broca/CH06 (*p* < 0.005); (b) left Wernicke/Ch14 to left Broca/Ch6 (*p* < 0.010); (c) left Wernicke/Ch14 to right Wernicke/Ch22 (*p* < 0.021). The first yellow circle represents the onset of the first trial over 500 ms, and the second one represents stimulus period presentation of the first trial over 2200-2700 ms integrating the semantic judgment; (d) causality strength across ROI (*p* < 0.05) in the meaningless condition during the experimental task.

Granger Prediction Estimation generates directional relations measured from HBO2 measurements among four significant ROIs (left Broca/Ch06, left Wernicke/Ch14, right Broca/Ch12, and right Wernicke/Ch22) in meaningless condition during the experimental task (Figure 6). Right Broca and left Wernicke present higher causality strength compared to other ROIs (^∗^*p* < 0.05). Time series causal relations are provided as the *x*-axis, and GPE is presented in the left column as the *y*-axis. A significant causal effect (*p* < 0.05) with higher causality strength is illustrated by a larger arrow, while the narrow indicator presents lower causality strength. A nonsignificant causal relationship (*p* > 0.05) is represented by the narrowest arrow, while the circle symbol illustrates the brain regions of interest (ROIs). The *t* value was presented to indicate the activation level elicited by each ROI.

## 4. Discussion and Conclusions

The current Granger Prediction Estimation (GPE) method investigated the causal mappings involved in Chinese figurative language comprehension recorded by haemodynamic responses that explained a meaningful/meaningless dichotomy. Our analyses revealed four significant haemodynamic changes located in frontal and temporal-parietal cortices, illustrating the nature of bilateral processing in figurative language. They also showed stronger causal relationships from the left to the right anterior frontal cortex capable of adjusting semantic processing into the plausible context and a bottom-up mechanism in the right hemisphere during the semantic integration of figurative language. Moreover, the interhemispheric directional flow over the anterior frontal cortex and bottom-up mechanism from the posterior temporal to anterior frontal cortex suggest, respectively, a key modulation of the right hemisphere capable of decoding novel literal sentences and mapping logographic-semantic structures. Finally, interhemispheric causal relationships found over the anterior frontal cortex and posterior temporal cortex suggest fine-grained integration activated by ambiguity and nonsense words. Also, the bottom-up mechanism reveals key circuits responsible for the storage and retrieval of semantic information in meaningless comprehension.

Relying on the main findings, the current study is aimed at proposing an effective connectivity map as a relevant tool to guide neural plasticity studies in language comprehension using Granger Prediction Estimation over time series captured by optical neuroimaging. Up until now, no effective connectivity studies were conducted to map causal relations during language comprehension tasks in Mandarin Chinese, as well as to solve limitations found in previous effective connectivity studies. More specifically, the resolution captured by near-infrared light has not been clearly supported by the frequency domain, which makes it difficult to conduct a precise analysis of the task-related event.

The computational strategy supports our causality hypothesis, providing detailed information about the brain network organization over time series resolution, during a language comprehension task. The brain networks of interest were first identified based on the activation found in the haemodynamic and brain imaging studies: Broca's area (left IFG/channel 6), its homolog (right IFG/channel 12) and right MTG (channel 22) (*p* < 0.05^∗^), and nonsignificant activation extensively supported by previous research [20] left Wernicke (Ch 14) in language comprehension tasks [22]. Secondly, the causal relations were examined over time series across different conditions to explore the agreement with the experimental task. The direction and strength of the causal relationships were investigated in the time domain, examining the onset time, stimulus presentation, and judgment period across all the significant channels detected on each region of interest (ROI). Significant causal directions were detected over 2200-2700 ms, which includes the onsite period (500 ms), stimuli period (2200 s), and blank screen/question mark (750 ms), confirming the causality hypothesis (*p* < 0.05^∗^) and revealing the good agreement with the stimulus processing during the comprehension task to guide neuromodulation intervention (*p* > 0.05). Finally, the brain networks were inspected through dynamic time series; however, the causality cannot be confirmed since nonsignificant causal relations were not detected.

In more detail, our main findings suggest that an increased bilateral activation with higher recruitment of the right hemisphere guides the network organization and explains how the networks influence each other during metaphor comprehension. Consistently, the directional flow from the right MTG to the right IFG and from the left IFG to the right IFG represent relevant mechanisms introduced by previous research work [4, 13] in line with our brain imaging study extracted from HbO measurements. A stronger causal relationship might reflect higher cognitive cost during the semantic judgment for novel metaphors. For those trials, stronger causal relations are required to coordinate the centre of memory retrieval by right MTG during the semantic judgment process. In addition, according to the conceptual mapping theory, the activation of sensorimotor cortices plays a crucial role in conceptualization required by Chinese metaphor processing [21], justifying the directional flow conducted from right MTG to right IFG, mainly when the source and target domains are processed.

Furthermore, the right anterior temporal network (ATN) has also been highlighted for distinguishing unfamiliar metaphors and the contribution of contextual semantic integration [5]. Concerning distinction skills, it was found that the pathway from left Broca's area (LIFG) to its homolog right (IFG) is crucial to distinguish figurative from literal processing, during the semantic integration. The influence of left Broca remains consistent during the onset and stimulus presentation for the first trial; however, right IFG seems relevant to guide the novelty versus familiarity processing during metaphor comprehension (time series 6000-7000 ms). A stronger causal relation generated by right IFG seems to appear when the salience categorization is not exact for the subject, guiding the novelty/familiarity distinction. Finally, the nonsignificant pathway observed in the left hemisphere from left Broca (left IFG) to left Wernicke (left MTG) highlights the higher-order cognitive function hub related to speech and language processing [17], such as working memory. Broca's area coordinates frontoparietal-temporal networks involved in semantic integration [20], which is consistent with a highly connected flow conducted from left IFG to left MTG. Furthermore, the left IFG has been highlighted as a memory retrieval centre to adjust semantic processing into the sentence context [5, 16]. Besides the highly connected hub between the two regions of interest, the causal relation did not play a significant role in explaining novel metaphor comprehension in Chinese Mandarin, as observed in the right hemisphere.

The recruitment of the right hemisphere during literal processing seems to not be exclusive to the metaphor comprehension, as shown in Figure 3. As for literal comprehension, the directional flow is also observed from the right MTG to right IFG, suggesting a bottom-up directional relationship due to the influence of spatial processing [22, 23] and logographic structure [21] on semantic integration. Therefore, it seems that the directional flow plays a vital role when shape recognition of the Chinese character is activated [24, 25]. Moreover, the causal relationship between the brain regions highlights the mechanism implied in the Wernicke-Geschwind model [26]. The neural pathway reports a visual directional flow coming from occipital to right MTG (“right Wernicke”) via arcuate fasciculus to right IFG area (“right Broca”), which seems to be responsible for the language motor abilities required by the sentence judgment. Furthermore, we found that causal flow from right to left Broca might be activated due to distant semantic relations and nonfamiliar literal sentences [27, 28]. The right hemisphere engagement supports the Gradient Salience Hypothesis proposed by Giora [28] which highlights that nonsalient meanings [29, 30] are processed firstly by the right hemisphere and guide the processing to reduce the ambiguity found in literal meanings [31]. In both directional relationships, the right hemisphere's contribution is observed during the semantic judgment peaks (interval of 2200 ms for the first trial), which echoes a solid agreement with the experimental task. The interhemispheric directional flow over the anterior frontal cortex and bottom-up mechanism from the posterior temporal to anterior frontal cortex suggest a key modulation of the right hemisphere capable of decoding unfamiliar literal sentences and mapping logographic-semantic structures able to store and retrieve semantic information.

As for the meaningless condition, the results propose an interhemispheric causal relationship from left Wernicke (LW) to its homolog, due to the fine-grained semantic integration traditionally guided by the left hemisphere. To be precise, the causal flows occur when the semantic cost is higher, such as during the stimulus duration and the peak of semantic judgment (2000-4000 ms for the first trial). Moreover, a significant causal relation from left MTG (Wernicke's area) to left IFG (Broca's area) was found, which proposes an engagement of the semantic storage hub managed by left MTG during the nonsense sentences towards the IFG retrieval centre. In line with a previous fMRI study [27], left IFC and left MTG were also activated to explain semantic processing in the Chinese language. Besides the role of earlier perceptive-attention skills to facilitate the semantic integration [20], IFG seems to be responsible for retrieving the semantic information stored by the left MTG. Finally, the significant causal relations also present a directional flow from right IFG to left IFG (Broca's area), confirming the key role of the right hemisphere when complex semantic structures [32], nonsense words, or sentences of difficult comprehension are processed (Figure 4).

Across all conditions, it is possible to observe neural pathways influenced by the Chinese logographic system. According to Bambini et al. [16], the directional flow conducted from right MTG to right IFG also reflects the critical role of the coordination hub located in the right MTG, associated with early motor functions and memory retrieval recruited by the semantic processing. Based on the evidence found, the computational strategy firstly confirms that the Chinese figurative language relies on the early top-down mechanism responsible for perceptive-attention engagement and the bottom-up mechanism underlying the semantic integration in both hemispheres as proposed in a previous study [29]. Furthermore, when interhemispheric connections are activated, the recruitment of the right hemisphere works to facilitate figurative comprehension, distant literal meanings, and reduce ambiguity during nonsense sentences. Finally, the findings suggest a good task agreement promoted by Granger Prediction Estimation to capture the causality strength across brain networks. The causal map clearly informs which brain regions and respective causal directions might be prioritized for the neuromodulation intervention, which enhances the protocol efficiency and minimizes the cognitive cost involved during brain training, such as tiredness and drowsiness side effects.

The main advantage of combining neuroimaging and Granger Prediction Estimation relies on examining the cognitive mechanism more deeply. However, the size of the population might be extended, and the methodology should also be applied to clinical populations with language disorders to improve the quality of diagnostic and treatment monitorization. In addition, by synthesizing multimodal imaging methods (e.g., ERP and fNIRS), both measurements helped to offer fine temporal resolution measures of neural activities (i.e., when) combined with cortical activations inferred by regional haemodynamic responses (i.e., where) [30], in which both can be complementary in revealing the neural mechanism of Chinese Mandarin language comprehension. Finally, the absence of meaningless answers should be added to counterbalance the yes/no dichotomy and addressed in future research work.

In conclusion, the current method proposes a brain network inspection conducted in temporal resolution captured by optical neuroimaging. GPE presents a reliable computational tool to investigate the complexity of human cognition, to target directional pathways for neuromodulation intervention in language comprehension and enhance the efficiency of clinical protocols in language disorders.

## Figures and Tables

**Table 1:** Sample characteristics.

Characteristics	*N* = 16	
Age (mean)^a^	24.5 years old	
Education (graduate, undergraduate)^b^	50%	50%
Gender (male, female)	50%	50%

^a^Age range (21-28 years). ^b^Graduate and undergraduate students recruited from the University of Macau.

**Table 2:** Behavioural results.

	Response accuracy^a^ (%)
Metaphor	0.577^b^
Literal	0.803^b^
Meaningless	0.143^b^

^a^ *F* (2, 30) = 19.271, *p* < 0.001. ^b^Standard deviation: metaphor 0.208, literal 0.143, meaningless 0.108.

**Table 3:** Anatomic labels.

Channels	Brodmann area	MNI
CH_1	Left temporal middle gyrus (BA 21)	-56 -1 -13
CH_2	Left temporal middle gyrus (BA 21)	51 0 6
CH_3	Left temporal middle gyrus (BA 21)	-50 -9 -10
CH_4	Left temporal middle gyrus (BA 21)	-54 -4 -3
CH_5	Left inferior frontal gyrus/pars opercularis (BA 44)	-59 6 11
CH_6	Left inferior frontal gyrus/rolandic operculum (BA 44)	-56 -1 10
CH_7	Right precentral gyrus (BA 4)	57 11 17
CH_8	Right precentral gyrus (BA 4)	70 6 17
CH_9	Right rolandic operculum (BA 44)	56 10 05
CH_10	Right superior temporal gyrus (BA 22)	49 4 -8
CH_11	Right superior temporal pole (BA 38)	66 4 2
CH_12	Right inferior frontal gyrus (BA 44)	48 0 -5
CH_13	Left inferior temporal gyrus (BA 21)	-39 -48 -10
CH_14	Left middle temporal gyrus (BA 21)	-41 -50 0
CH_15	Left fusiform (BA 37)	-32 -62 -5
CH_16	Left middle temporal gyrus (BA 21)	-49 -73 7
CH_17	Left middle occipital gyrus (BA 19)	43 -55 24
CH_18	Left fusiform (BA 37)	-38 70 27
CH_19	Right superior temporal gyrus (BA 22)	45 -42 18
CH_20	Right middle temporal gyrus (BA 21)	45 42 50
CH_21	Right middle temporal gyrus (BA 21)	45 -58 22
CH_22	Right middle temporal gyrus (BA 21)	60 -50 1
CH_23	Right middle temporal gyrus (BA 21)	61 -49 -10
CH_24	Right inferior temporal gyrus (BA 20)	42 -59 -8

## Data Availability

The fNIRS data that support the findings of this study are available from the corresponding author, upon request.
